# Idiotype/anti-idiotype antibodies: as a glorious savior in COVID-19 pandemics

**DOI:** 10.1186/s41231-021-00097-y

**Published:** 2021-08-23

**Authors:** Ahsan Naveed, Deeba Naz, Sajjad ur Rahman

**Affiliations:** 1 Chonnam National University, Gwangju, South Korea; 2 University of Agriculture Faisalabad, Faisalabad, Pakistan

**Keywords:** COVID-19, SARS-2, Idiotype, Anti-idiotype, Vaccine

## Abstract

The idiotype network is experimentally modified to provide protective immunity against various microbial pathogens. Both internal and non-internal image-idiotype antibodies can trigger specific immune responses to antigens. The current outbreak of Severe Acute Respiratory Syndrome 2 (SARS-2) has provided a great opportunity to take advantage of idiotype / anti-idiotype antibodies as a protective regimen when no approved vaccine is available on earth. The current review identifies successful applications of idiotype/ anti-idiotype antibodies in various viral diseases and highlights their importance in COVID-19 pandemics. In the absence of vaccines and targeted therapies, polyclonal idiotype/ anti-idiotype antibodies against the viral structure may be a potential approach to the prevention and treatment of COVID-19 patients.

## SARS Coronavirus the escaped killer

SARS-CoV‐2, a member of the Coronaviridae family of the order Nidovirales are enveloped, positive‐sense, single‐stranded, and highly diverse RNA virus [1]. It has a crown-like shape. The genome is 26–32 Kb in length and is considered highly pathogenic [2, 3]. This family is subdivided into coronavirinae with four major generas such as alphacoronavirus, beta coronavirus, gamma coronavirus, delta coronavirus [4]. Viruses included in the alphacoronavirus and beta corona viruses affect most mammals, while the gamma coronavirus affects avian species. The delta coronavirus genus is found in both mammalian and avian hosts [5, 6]. The virus was reported in the Chinese province of Wuhan and is now classified as an pandemic. [7, 8]. They can cause infections in the gastrointestinal, respiratory, liver, and central nervous systems of humans, cattle, birds, bats, and many other wild animals [9–11]. Studies have shown strong evidence of close-knit among acute respiratory syndrome corona virus (SARS-CoV) and Middle East respiratory syndrome corona virus (MERS-CoV) [12–16].

## Virus host interaction

Corona viruses are spherical as shown by Cryo-electron microscopy [17]. Corona virus particles contain four main structural proteins, including spike (S), membrane (M), envelope (E), and nucleocapsid (N) proteins. These structural proteins are necessary for virulence assembly and infection of CoVs. S proteins form spikes at the viral surface and are responsible for attachment to host receptors [18, 19]. Transmembrane domains of more prevalent viral M protein are involved in membrane curvature and budding [20, 21] It has critical role in giving shape to the virions, and binds to the nucleocapsid [22]. Viral E protein has diverse functions from assembly to the egress and interactions with host cell [23, 24]. The dynamic functions of coronavirus N proteins are involved in the formation of replication transcription complexes [25]. The initial attachment to the host cell is through interaction between the S protein and its receptor. Virion particles enter into the host cell *via* binding through the Angiotensin-converting enzyme 2 (ACE2). Subsequently, the coronavirus genome (ss RNA) attaches to the host’s ribosomes leading to the translation of 2 *co*-terminal and large polyproteins further processed by proteolytic enzymes [26, 27]. Proteolysis results in the smaller components for folding and packaging of new virions. These new virions bud through the intracellular membranes and are later released from the cells through vesicles of the secretory pathway [28, 29].

## Humoral immune response to viral attack

Viral invasion leads to the development of either innate or adaptive immunity. Innate immunity is associated with the recognition of pathogen-associated molecular patterns (PAMPs) causing cytolysis, through the natural killer cells and the interferons causing the death of virus along with its resident cell. Adaptive immunity, on the other hand, plays a vital role in clearing the virus either through the cell-mediated immunity by cytotoxic T cells including CD8 + and the CD4 + subset, that kill the virus-infected cells or through the production of the B cells with antibody as a major arm [30].

The humoral immune system gives rise to natural antibodies secreted by plasma cells upon an initial viral infection [31]. B lymphocytes are stimulated upon viral infection and differentiate into plasma cells to generate antibodies. An antibody can result in the neutralization of the virus either by hindering virus-host cell interactions or through the recognition of viral antigens on infected cells leading directly to the antibody-dependent cytotoxic cells (ADCC) or complement-mediated lysis. Mostly, IgG antibodies are responsible for the antiviral activity, while IgA is prevalent when viral infection occurs at the mucosal surfaces [32]. In vitro studies have revealed that protection through antibodies is conferred by neutralization using an infectious virus and susceptible cells [33, 34]. Neutralizing antibodies are therefore essential for the protection from viral infection [35] by targeting viral glycoproteins of enveloped viruses or the protein coat of nonenveloped viruses [36].

## Antigenic mimicry of viral structure

Antigen-specific approaches in immunotherapy have gained much attention with experimental animals showing promising results upon antigen-based treatments leading to improvement in disease status [37]. The immunoglobulins are glycoprotein in nature with two identical heavy and light chains. These heavy-light chains comprise of two variable (V) immunoglobulin (Ig) domains (V_L_ and V_H_) that result in a unique surface for antigen binding and these two V domains culminate the generation of the idiotype (Id) [38]. The term idiotype (Id) denotes an entire collection of idiotopes present in a single immunoglobulin (Ig) molecule and develop because of somatic mutations. Anti-idiotype (Anti-Ids) antibody, on the other hand, attaches to the idiotypic variable domains of an antibody [39]. Idiotopes can be regarded as foreign because the tiny amount of them normally present in any individual is inadequate to elicit self-tolerance and hence can be immunogenic [40]. The binding site of idiotype (Ab1) antibodies imitates the original antigen and give rise to anti-idiotype antibody (Ab2) mimicking the complete internal image of the antigen [41] as well as displays a functional activity that looks like the natural physiological activity of the antigen [42].

Idiotype are picked up by antigen-presenting cells (APCs) such as macrophages, dendritic cells, and B lymphocytes, and are then presented to T cells on MHC class II molecules. B lymphocytes continuously present Id-peptides on MHC class II molecules [43, 44]. The introduction of these identifiable peptides leads to the generation of anti-idiotypes. This presentation initiates a signal for B-lymphocytes to differentiate between antibody-producing plasma cells and secrete anti idiotype antibodies [45, 46]. B lymphocytes secrete antibodies that bind to their B cell receptor (BCR). Consequently, anti-Ids are introduced into MHC class II molecules [47]. This interaction between T and B cells produces antigen-free cooperation between T cells and B cells, which is called Id-driven TB cell cooperation, resulting in constant activity of both T and B cell in the absence of antigen [48]. This antigen-free mechanism is important for the production and upkeeping of immunological memory in the absence of antigen [49, 50]. It has been suggested that polyclonal idiotypic determinants are shared between B and T cells for the same antigen. However, monoclonal antibodies have not given these results due to the reason that BCR binds to the native epitopes whereas TCR can bind the antigens once they have been processed and presented on MHC molecules by the antigen presenting cells [51].

## Current therapeutic and prophylactic approaches to COVID19

Vaccine development is a lengthy and costly process, and it takes years to develop a successful licensed vaccine [52]. Multiple techniques have been in use to prevent infectious diseases including conventional and modern vaccine pathways. DNA and RNA-based vaccines are developed rapidly as there is no prior need for the culture [53]. The development of the SARS-CoV 2 vaccine outpaced all the previous efforts resulting in over 300 projects of vaccine development in few months [54]. Currently, approved vaccines for SARS-CoV2 include inactivated (Sinopharm and SinoVac), vector-based (CanSinoBio, Sputnik V, and AstraZeneca), and mRNA-based vaccines (Pfizer BioNTech and Moderna). Inactivated SinoPharm and SinoVac vaccines both manufactured in China and studies have demonstrated that they are immunogenic and safe [55]. CanSinoBio comprises a single dose jab incorporated in an adenovirus vector and has gained much attention as the first choice for use in emergency cases including Pakistan and Mexico whereas trials are also underway in Argentina and Russia [56]. Russia’s Sputnik V vaccine carries the mRNA for spike protein of Coronavirus in two human adenoviruses vectors which are given in two different doses whereas Oxford–AstraZeneca vaccine makes use of similar material for both doses (chimpanzee adenovirus vectored vaccine ChAdOx1 nCoV-19) [57, 58]. Pfizer BioNTech and Moderna vaccines make use of mRNA-based approaches which is present in liposomes and can produce antigen proteins coded by mRNA [59].

Besides vaccines, various therapeutic interventions have been tested to cure COVID 19 patients including Hydroxychloroquine, Azithromycin, and Remdesivir. Hydroxychloroquine has been found to interfere with the glycosylation of ACE2 resulting in the prevention of virus binding [60, 61]. Azithromycin in synergism with Hydroxychloroquine has suggested rapid viral clearance [62] however, this data is not sufficient to conclude any authentic results. Moreover, high doses of Hydroxychloroquine are also not suggested in severely ill COVID19 patients [63]. Remdesivir, an analog of nucleoside, can inhibit viral RNA-dependent RNA polymerase activity [64] and was successfully used in a SARS-CoV2 infected individual [65]. Its side effects are minimal resulting in decreased mortality.

## Idiotypes as Pandemic savior

In human history, COVID-19 is the second major respiratory challenge after influenza. Influenza virus threatens global health despite efforts to develop an effective vaccine [66]. Being an RNA virus, replication results in mutations of SARS-CoV2 naturally. To date, 4000 known mutations have been reported in the spike protein region [67]. Antibodies are the key immune cells that confer the protective response to various pathogens [68]. The durability of antibodies depends upon the virus type as well as host and environmental factors [69, 70]. Memory B cells are involved in recall responses whereas plasma cells are only the source of circulating antibodies. Studies have revealed that memory B cells display somatic hypermutation in SARS-CoV2 indicating the continuous evolution of humoral immune response [71]. Moreover, it has been found that the immunity to seasonal coronaviruses is short-lived [72]. Reports are that newer mutations in the SARS-CoV2 may lead to immune escapes resulting in the reinfection and updating the existing vaccines. However, it has been found that the mutations in virus have not caused any vaccines upgradation making the immune system memory effective against SARS-Cov2 virus [73].

Even the development of the Covid 19 vaccine is still in its infancy, with the need to develop a universal vaccine against several antigenically different viruses, including those currently in circulation and those may come to light in the future. The development of idiotype vaccines may reduce the risk of unwanted side effects that are usually produced from the most common antigenic vaccines [74]. Idiotype vaccines are proteins in nature and can be easily handled. They are T cell-dependent antigens, therefore, effective immunogenic carriers can be used as agents to pair with the antigen. [75]. Studies have shown that immunization of BALB/c mice with pure chicken anti-H9 IgG promotes the development of anti-idiotypic antibodies and specific B-cell hybridomas. After screening for hybridomas, a monoclonal antibody was identified that was able to bind hemagglutinin and produce antibodies [76].

Antibodies may be of the IgM, IgG, and IGA types that are directed to the spike (S) protein of SARS-CoV2. Idiotype is a sequence switch of antibodies that attaches to the receptor binding domain (RBD) of the viral S protein. The initial antibody belongs to the IgM isotype which later changes to the IgG isotype. Class switching can also occur with the IgA isotype, which is produced by large-scale plasma cells in the laminar propria of mucosal surfaces. IgA antibodies play an important role in the immune defense of the virus, the entry point of the virus to the mucosal surface of the respiratory tract, related to the formation of an important immunoglobulin. IgA can neutralize SARS-CoV 2 before it reaches the epithelial cells and attaches to them. IgA may play a role in a mucosal vaccine to promote the development of idiotypic vaccines [48]. Previous research has also shown that the anti-idiotype vaccine can elicit mucosal immunity and act as an immunogenic against the coronavirus [77].

In severe SARS-CoV 2 infections, binding to different cytokines or variable domains of other antibodies (anti-idiotype antibodies) is thought to reduce the inflammatory response. Furthermore, the presence of IgG dimers may interfere with the activation of FcγR on innate immune cells [78]. It has been speculated that high doses of anti-idiotypic immunoglobulins may be helpful in severe SARS-Co-2 infections by modulating the immune system, centralizing FcγR, and lowering ADE [79].

## Conclusions

The biggest challenge today is the COVID-19 pandemic. At present, more than 2.69 million people have been killed worldwide and about 122 million positive cases have been reported. Vaccine and effective treatment strategies are growing rapidly, but time is needed to make effective progress. Significant life-saving support was offered in the treatment of plasma in patients with COVID 19. Considering past and present research, it has been pointed out that neutralizing antibodies in the form of hyperimmune serum has always been valuable in saving lives. Timely implementation of controlled studies on immunoglobulins for the development of idiotype / anti-idiotype vaccines is needed.

## Data Availability

Not Applicable.

## Abbreviations

ADE: Antibody dependent enhancement
FcγR: Fc gamma receptor
ssRNA: Single stranded RNA
